# Non‐water‐suppressed short‐echo‐time magnetic resonance spectroscopic imaging using a concentric ring *k*‐space trajectory

**DOI:** 10.1002/nbm.3714

**Published:** 2017-03-08

**Authors:** Uzay E. Emir, Brian Burns, Mark Chiew, Peter Jezzard, M. Albert Thomas

**Affiliations:** ^1^ FMRIB Centre, Nuffield Department of Clinical NeurosciencesUniversity of Oxford, John Radcliffe HospitalOxfordUK; ^2^ Department of OncologyUniversity of OxfordOxfordUK; ^3^ Department of Radiological SciencesUniversity of CaliforniaLos AngelesCAUSA

**Keywords:** concentric rings, metabolite‐cycling, non‐water‐suppressed, spectroscopic imaging

## Abstract

Water‐suppressed MRS acquisition techniques have been the standard MRS approach used in research and for clinical scanning to date. The acquisition of a non‐water‐suppressed MRS spectrum is used for artefact correction, reconstruction of phased‐array coil data and metabolite quantification. Here, a two‐scan metabolite‐cycling magnetic resonance spectroscopic imaging (MRSI) scheme that does not use water suppression is demonstrated and evaluated. Specifically, the feasibility of acquiring and quantifying short‐echo (*T*_E_ = 14 ms), two‐dimensional stimulated echo acquisition mode (STEAM) MRSI spectra in the motor cortex is demonstrated on a 3 T MRI system. The increase in measurement time from the metabolite‐cycling is counterbalanced by a time‐efficient concentric ring *k*‐space trajectory. To validate the technique, water‐suppressed MRSI acquisitions were also performed for comparison. The proposed non‐water‐suppressed metabolite‐cycling MRSI technique was tested for detection and correction of resonance frequency drifts due to subject motion and/or hardware instability, and the feasibility of high‐resolution metabolic mapping over a whole brain slice was assessed. Our results show that the metabolite spectra and estimated concentrations are in agreement between non‐water‐suppressed and water‐suppressed techniques. The achieved spectral quality, signal‐to‐noise ratio (SNR) > 20 and linewidth <7 Hz allowed reliable metabolic mapping of five major brain metabolites in the motor cortex with an in‐plane resolution of 10 × 10 mm^2^ in 8 min and with a Cramér‐Rao lower bound of less than 20% using LCModel analysis. In addition, the high SNR of the water peak of the non‐water‐suppressed technique enabled voxel‐wise single‐scan frequency, phase and eddy current correction. These findings demonstrate that our non‐water‐suppressed metabolite‐cycling MRSI technique can perform robustly on 3 T MRI systems and within a clinically feasible acquisition time.

**Abbreviations used**
2D: two dimensional
Cho: choline
Cr: creatine
CRLB: Cramér‐Rao lower bound
EPSI: echo‐planar spectroscopic imaging
FFT: fast Fourier transform
FOV: field of view
GABA: γ‐aminobutyric acid
Gln: glutamine
Glu: glutamate
GM: grey matter
GPC: glycerophosphocholine
HLSVD: Hankel‐Lanczos singular value decomposition
Lac: lactate
MRSI: magnetic resonance spectroscopic imaging
*myo*‐Ins: *myo*‐inositol
NAA: N‐acetylaspartate
NUFFT: non‐uniform FFT
OVS: outer volume suppression
PE: phosphoethanolamine
SAR: specific absorption rate
SD: standard deviation
SNR: signal‐to‐noise ratio
STEAM: stimulated echo acquisition mode
SV: single voxel
tCho: total choline
tCr: total creatine
tNAA: total N‐acetylaspartate
VAPOR: variable‐power RF pulses with optimized relaxation delays
VOI: volume of interest
WM: white matter

## 1 INTRODUCTION

Non‐invasive measurement of metabolite concentrations by proton MRS is of great potential value for studying the metabolic state of healthy and diseased brains.^1^ For example, MRS allows detection of a variety of neurochemicals, including N‐acetylaspartate (NAA) as a marker of neuronal loss/dysfunction, creatine (Cr) as a marker for deficits in energy metabolism, choline (Cho) as a marker for cell membrane turnover, glutamate (Glu) as the primary excitatory neurotransmitter and γ‐aminobutyric acid (GABA) as the primary inhibitory neurotransmitter. Thus, MRS can contribute not only to the diagnosis and monitoring of disease,^1^ but also to the measurement of modulations in functional neurochemistry during physiological interventions.^2^ For example, the detection of NAA and GABA has been valuable in helping to understand neuropathological and biochemical abnormalities in neurodegenerative diseases such as amyotrophic lateral sclerosis^3^ and Parkinson's disease.^4^ In addition, non‐invasive detection of the oncometabolite 2‐hydroxyglutarate in brain tumours using MRS has the potential to be an imaging biomarker to monitor disease progression and response to therapy.^5^, ^6^, ^7^

MRS data can be obtained either from a single voxel (SV‐MRS), albeit limited to a small volume of interest (VOI), or from multiple voxels (magnetic resonance spectroscopic imaging, MRSI), which acquires metabolic profiles over larger regions of the brain. However, compared with SV‐MRS, the use of MRSI has been limited by several challenges, such as inhomogeneity of the main (*B*_0_) and RF magnetic fields, long acquisition times, insufficient water suppression, eddy‐current‐induced gradient errors, and line broadening artefacts caused by subject motion and scanner instability.^1^

In order to separate metabolite signals from abundant water signal robustly, *in vivo* MRS methods require techniques for suppression of the water signal during acquisition^8^, ^9^, ^10^, ^11^ and/or post‐processing.^12^ The vendor‐provided MRS packages on clinical scanners offer water‐suppressed spectroscopic acquisition techniques as the standard approach.^13^ However, the acquisition of a non‐water‐suppressed MRS spectrum is generally required to act as an internal reference for metabolite quantification. This information can also be required for optimal reconstruction of spectra from different phased‐array coils, the correction of gradient‐induced sideband modulations, eddy‐current‐induced artefacts and tracking *B*_0_ drifts due to subject motion or scanner drift. Although numerically optimized water suppression techniques are available,^9^ vendor‐provided MRS packages on clinical scanners are not fully optimized and result in, for example, baseline distortions due to poor water suppression. The recent advances in MRI hardware, especially the increase in the effective dynamic range of the analogue‐to‐digital converter (>12 bit), enable us to acquire water and metabolite signals simultaneously. Thus, *in vivo* MRS techniques (and particularly MRSI) that do not require water suppression^12^, ^13^, ^14^, ^15^, ^16^, ^17^, ^18^ can address this problem by eliminating the requirement for the water suppression whilst inherently providing acquisition of the water reference spectra.

In recent years, fast *k*‐space trajectories, both echo‐planar (echo‐planar spectroscopic imaging or EPSI^19^) and non‐echo‐planar (spiral,^20^ rosette^21^ and concentric^22^, ^23^, ^24^ spectroscopic imaging), have been developed to reduce the number of required phase encoding steps and, therefore, the acquisition time. It has been demonstrated that MRSI using an EPSI trajectory enables 3D metabolite mapping with whole brain coverage within clinically acceptable acquisition times.^25^, ^26^, ^27^ Although EPSI reduces acquisition duration in one spatial direction, it still suffers from relatively long scan times due to the number of required phase encoding steps. Alternatively, spiral and rosette *k*‐space trajectories offer reduced acquisition duration for MRSI but suffer from discrepancies between their desired and true trajectories due to system imperfections (such as gradient timing delays and eddy currents). The concentric rings trajectory, which uses concentrically circular trajectories, is time efficient compared with EPSI and is less sensitive to system imperfections compared with spiral MRSI.^22^ However, non‐uniform *k*‐space sampling results in signal‐to‐noise ratio (SNR) loss.^22^

The intent of this work, therefore, is to develop a non‐water‐suppressed MRSI acquisition technique that addresses the challenges of sensitivity, spectral quality, speed and spatial resolution whilst also providing the required information for voxel‐wise single‐scan frequency, phase, gradient‐induced sideband and eddy current correction. To achieve this, a non‐water‐suppressed metabolite‐cycling two‐dimensional (2D) MRSI acquisition technique with asymmetric narrow‐transition‐band adiabatic inversion pulses^14^, ^15^, ^16^ is proposed for simultaneous detection of the metabolites and water signals at short echo time (*T*_E_ = 14 ms) using stimulated echo acquisition mode (STEAM) localization. Even though a major drawback of this method is due to the doubling of the measurement time by the metabolite‐cycling, it is counterbalanced by a time efficient concentric ring *k*‐space trajectory. To validate metabolite profiles obtained using the newly developed acquisition technique, we compare profiles quantified from non‐water‐suppressed and conventionally water‐suppressed STEAM MRSI scans acquired from the motor cortex.

## 2 METHODS

Five healthy volunteers (three males/two females, aged 28.8 ± 3.4 (mean ± sd) years) participated in this study after giving informed consent under an institutionally approved technical development protocol.

### 2.1 MRI data acquisition

All scans were acquired using a Siemens Prisma 3‐Tesla (Siemens, Erlangen, Germany) whole body MRI scanner (with a maximum gradient of 80 mT/m and maximum slew rate of 200 mT/m/ms) and a 32‐channel (*N*_cha_) head array receive coil. A high‐resolution *T*_1_‐weighted MP‐RAGE dataset (*T*_R_ = 1900 ms, *T*_E_ = 3.97 ms, *T*_I_ = 904 ms, flip angle =8°, 192 transverse slices, 1 mm^3^ isotropic voxels) was also acquired for accurate MRSI grid placement. *B*_0_ shimming was achieved using GRESHIM (gradient‐echo shimming).^28^

### 2.2 MRSI data acquisition

#### 2.2.1 Non‐water‐suppressed metabolite‐cycling MRSI

**Figure 1:**
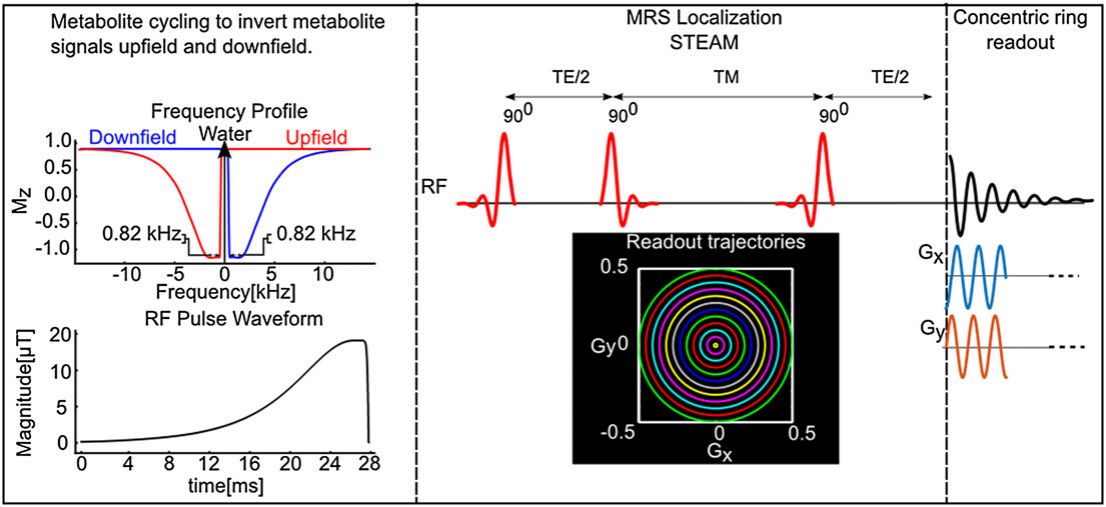
Pulse sequence diagram of the proposed MRSI method without water suppression, allowing simultaneous detection of metabolite and water signals by two scans. Prior to a STEAM localization, two asymmetric narrow‐transition‐band adiabatic RF pulses invert the spectral range where metabolite signals are expected either upfield or downfield with respect to water. Instead of a conventional *k*‐space trajectory, the preparation scheme is followed by a concentric ring *k*‐space trajectory

The non‐water‐suppressed metabolite‐cycling MRSI acquisition was achieved by utilizing two asymmetric narrow‐transition‐band adiabatic RF pulses with mirrored inversion profiles applied in alternate scans for the inversion of the upfield and downfield (relative to water) spectral resonances before the STEAM localization with a gap of 9.6 ms.^14^ Using a maximum *B*_1_ of 19 μT and pulse duration (*T*_p_) of 27 ms, an 80 Hz transition bandwidth (−0.95 < *M*_*z*_/*M*_0_ < 0.95) and 820 Hz inversion bandwidth (−1 < *M*_*z*_/*M*_0_ < −0.95, 70 to −750 Hz) downfield/upfield from the carrier frequency was achieved. The centre of the transition band (*M*_*z*_ = 0) was placed at the carrier frequency offset by +60 Hz and −60 Hz for downfield and upfield, respectively. Figure 1 shows the proposed pulse sequence (adiabatic pulse parameters: hyperbolic secant pulse, HS_1/2_, with *R* = 10 and 0.9 × *T*_p_, tanh/tan pulse with *R* = 40 and 0.1 × *T*_p_).^29^

#### 2.2.2 Water‐suppressed MRSI

For comparison, a water‐suppressed MRS acquisition was also made by utilizing an outer volume suppression (OVS) and VAPOR (variable‐power RF pulses with optimized relaxation delays) water suppression scheme before the STEAM localization.^30^, ^31^ A Gaussian RF pulse (20.48 ms duration) and crusher gradient (6.6 ms) were used during the mixing time (*T*_M_) period of STEAM to suppress residual water signal in addition to the VAPOR water suppression. Eight RF pulses with variable pulse power and optimized timing were used for VAPOR water suppression. To suppress unwanted signals outside the VOI, OVS pulses were also applied as described in Reference 30.

#### 2.2.3 *In vivo* MRSI acquisitions

All *in vivo* 2D MRSI scans from a specific region of interest in the motor cortex were manually positioned using the high‐resolution *T*_1_‐weighted MP‐RAGE image. STEAM localization (*T*_R_ = 2 s, *T*_E_ = 14 ms, *T*_M_ = 32 ms, number of averages, *N*_avg_ = 20) was used to excite a 65 mm × 65 mm × 20 mm region centrally within the field of view (FOV). The imaging box was localized with an FOV of 240 mm × 240 mm and a slice thickness of 20 mm. The 2D concentric *k*‐space trajectory was used to sample polar *k*‐space data.^23^ In order to minimize any reconstruction artefacts for the azimuthally undersampled polar *k*‐space data, the azimuthal sampling criterion, *N*_p_ring_ ≥ *πN*_ring_, was used, where *N*_p_ring_ is the number of points per ring in the range 0 ≥ *θ* ≥ 2*π* and *N*_ring_ is the number of rings.^32^ Thus, 64 points per ring (number of points per ring, *N*_p_ring_ = 64) were collected with an ADC bandwidth of 80 kHz and a maximum slew rate = 67.8 mT/m/ms. 512 temporal points were collected in an effective spectral bandwidth of 1250 Hz. The 64 points per ring were sufficient to satisfy the requirement of avoiding azimuthal aliasing.^32^ To cover the 24 × 24 grid in a minimum total acquisition duration, 12 rings (number of rings, *N*_ring_ = 12) resulting in an individual voxel size of 2 mL were acquired in 8 min (*N*_ring_ × *N*_avg_ × *T*_R_ = 480 s).^23^ This corresponded to a fully excited 6 × 6 voxel region with a ½ voxel margin outside the STEAM localization.

In order to demonstrate the potential advantages of the non‐water‐suppressed acquisition scheme for voxel‐wise single‐scan frequency alignment, as well as phase and eddy current correction, we conducted *in vivo* non‐water‐suppressed and water‐suppressed MRSI measurements from a specific region of interest in the motor cortex with a high in‐plane resolution of 5 mm × 5 mm × 20 mm (*N*_rings_ = 24, *N*_p_ring_ = 64, FOV = 240 mm × 240 mm, STEAM localization =80 mm × 100 mm × 20 mm, *T*_R_ = 1.5 s, *T*_E_ = 14 ms, *T*_M_ = 32 ms, *N*_avg_ = 20, ADC bandwidth =80 kHz, maximum slew rate = 168.2 mT/m/ms, *N*_ring_ × *N*_avg_ × *T*_R_ = 720 s) in a healthy subject. In order to simulate frequency changes due to patient motion and long‐term magnetic field drift, during *in vivo* MRSI measurements, transmit and receive frequency offsets were varied from 0 Hz to 18 Hz with a 2 Hz increment for every other MRS image. Although this *k*‐space sampling scheme starts to violate the azimuthal sampling criterion at the 20th ring, we did not observe any error in image quality, SNR or resolution (Supporting Figure S1).

#### 2.2.4 Phantom experiments

The two MRSI sequences were tested on two phantoms. First, the concentric ring *k*‐space trajectory and its reconstruction was tested on a cylindrical resolution phantom (General Electric Medical Systems, Milwaukee, WI, USA) using the non‐water suppressed metabolite‐cycling MRSI sequence with the following parameters: *N*_rings_ = 32, *N*_p_ring_ = 160, FOV = 320 mm × 320 mm, STEAM localization =160 mm × 160 mm × 10 mm, *T*_R_ = 2 s, *T*_E_ = 14 ms, *T*_M_ = 32 ms, *N*_avg_ = 2, ADC bandwidth =200 kHz, maximum gradient slew rate = 141.3 mT/m/ms. The 160 points per ring were sufficient to satisfy the requirement of avoiding azimuthal aliasing.^32^ Second, a phantom experiment for metabolite spectra depiction for both a water‐suppressed and the metabolite‐cycling technique was performed on an MRS ‘braino’ phantom (General Electric Medical Systems, Milwaukee, WI, USA) containing 10 mmol Cr, 3 mmol Cho, 5 mmol lactate (Lac), 1 mL/L Gd‐DPTA (Magnevist), 12.5 mmol Glu, 7.5 mmol *myo*‐inositol (*myo*‐Ins), 12.5 mmol NAA, 0.1% sodium azide, 56 mmol sodium hydroxide and 50 mmol potassium phosphate monobasic.

### 2.3 Post‐processing

All the reconstruction algorithms were implemented in MATLAB (MathWorks, Natick, MA, USA). Density compensation was applied to the non‐Cartesian *k*‐space data to grid it onto Cartesian *k*‐space,^33^ and then a fast Fourier transform (FFT) was performed. Gridding and FFT steps were done by using the non‐uniform FFT (NUFFT) toolbox with min‐max Kaiser‐Bessel kernel interpolation and twofold oversampling.^34^ The final matrix size of the reconstructed MRSI image after NUFFT was 2*N*_ring_ × 2*N*_ring_ × *N*_sp_ × *N*_cha_ × *N*_avg_.

#### 2.3.1 Metabolite‐cycling

Odd (upfield) and even (downfield) single‐shot non‐water‐suppressed FIDs edited by the asymmetric RF pulses were frequency and phase corrected on the basis of the water signal. The frequency correction was performed using a cross‐correlation algorithm and phase correction was performed using a least‐squares fit algorithm. Then, upfield (*S*_a_) and downfield (*S*_b_) edited spectra were summed and used for removing residual eddy current effects,^35^ combining the phased‐array coil spectra^36^ and metabolite quantification. Metabolite spectra were then calculated by subtraction of alternating FIDs. Potential differences in the water peak amplitude between upfield (*S*_a_) and downfield (*S*_b_) edited spectra were calculated, and then corrected to minimize the residual water peak in the final subtracted metabolite spectrum.^14^

(1) $$S_{\text{diff}}=S_{a}-k\times S_{b.}$$

The correction factor, *k*, was determined from the integral of the water peak.

#### 2.3.2 Water signal calibration for water‐suppressed MRSI

An additional unsuppressed water scan was acquired for the water‐suppressed MRSI protocol to remove residual eddy current effects^35^ and to combine the phased‐array coil spectra.^36^ Single‐shot metabolite spectra were then frequency and phase corrected prior to averaging over *N*_avg_ using a cross‐correlation and a least‐squares fit algorithm, respectively. No additional spatial smoothening was used.

#### 2.3.3 Metabolite quantification

The averaged metabolite spectrum was quantified using the LCModel package.^37^ The residual water peak was filtered with the Hankel‐Lanczos singular value decomposition (HLSVD) algorithm prior to the LCModel analysis.^38^ Concentrations were calculated using the unsuppressed water spectrum as an internal reference for *in vivo* data, whereas concentrations were reported relative to Cr for the phantom measurement. The model spectra of alanine (Ala), aspartate (Asp), ascorbate/vitamin C (Asc), glycerophosphocholine (GPC), phosphocholine (PC), Cr, phosphocreatine, GABA, glucose, glutamine (Gln), Glu, glutathione, Lac, *myo*‐Ins, NAA, N‐acetylaspartylglutamate, phosphoethanolamine (PE), *scyllo*‐inositol and taurine were generated based on previously reported chemical shifts and coupling constants^39^, ^40^ by the GAMMA/PyGAMMA simulation library of VeSPA (Versatile Simulation, Pulses and Analysis) to carry out the density matrix formalism.^41^ Simulations were performed using the same RF pulses and sequence timings as those on the 3 T system in use. Eight LCModel‐simulated macromolecule resonances were included in the analysis at the following positions: 0.91, 1.21, 1.43, 1.67, 1.95, 2.08, 2.25 and 3 ppm.^42^ Concentrations were not corrected for *T*_1_ and *T*_2_ effects or cerebrospinal fluid contribution. If the correlation between two metabolites was consistently high (correlation coefficient < −0.3) in a given region, their sum was reported, e.g. total creatine (Cr + PCr, tCr), total choline (GPC + PC, tCho) and Glu + Gln.

#### 2.3.4 Bland‐Altman analysis

To evaluate any discrepancies between non‐water‐suppressed and water‐suppressed techniques and the limits of agreement, a Bland‐Altman analysis^43^ was performed on those brain metabolites that had Cramér‐Rao lower bound (CRLB) goodness of fit values smaller than 20% (total N‐acetylaspartate (tNAA), tCr, tCho, *myo*‐Ins and Glu + Gln). For each subject and each reported metabolite in the voxels within the STEAM localization (a grid of 6 × 6), the difference (in μmol/g) between the two techniques was calculated. Assuming that the differences between techniques are approximately normally distributed, 95% of the differences will fall between plus or minus 1.96 standard deviations (SD) of the mean difference (the limits of agreement or Bland‐Altman reproducibility coefficient).

## 3 RESULTS

### 3.1 Phantom measurements

**Figure 2:**
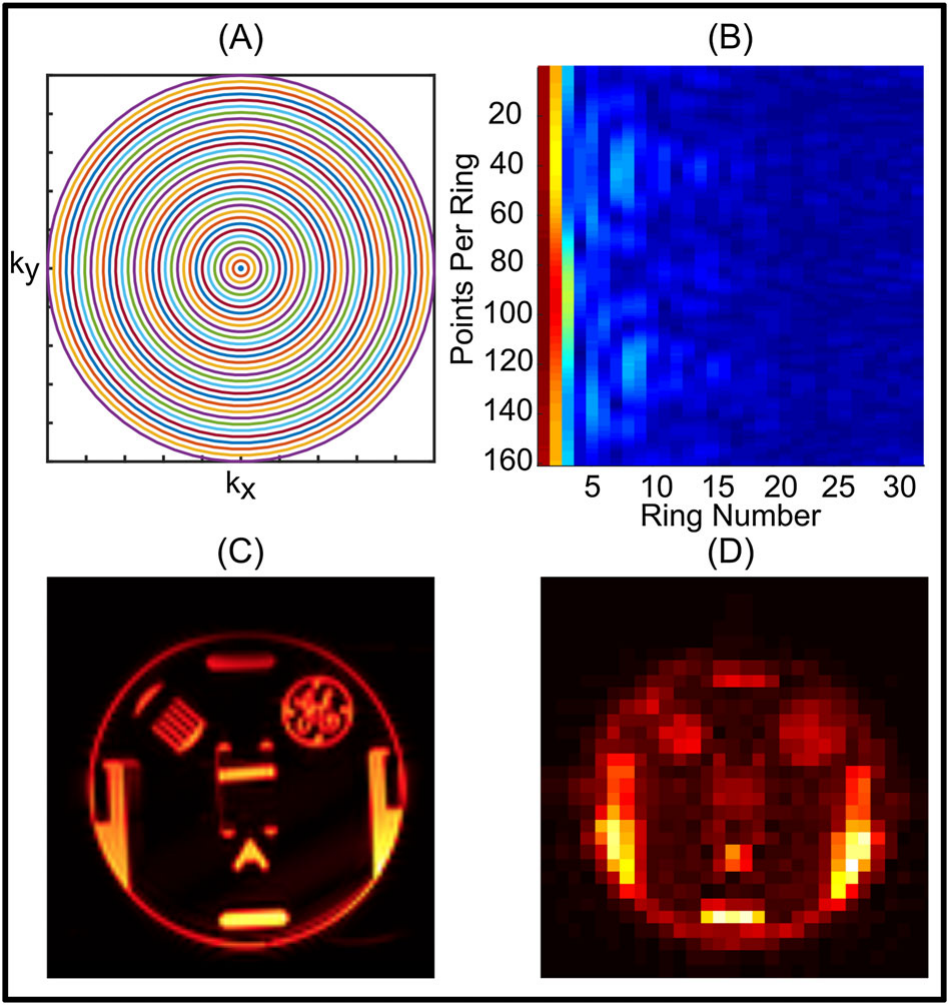
A, Concentric ring *k*‐space trajectory used for the resolution phantom experiment. B, *k*‐space data acquired using non‐water‐suppressed metabolite‐cycling MRSI. C, High‐resolution *T*_1_‐weighted MPRAGE image of the slice studied. D, Water image with a final grid of 64 × 64 (2*N*_ring_ × 2*N*_ring_) obtained using the first time point of the water FID

Figure 2 shows the results from the resolution phantom for the non‐water‐suppressed metabolite‐cycling MRSI technique, as well as the concentric ring *k*‐space trajectory used, along with the resulting *k*‐space data. Figure 2C shows the conventional MP‐RAGE image, and Figure 2D demonstrates the image derived from the MRSI data. Although the final resolution of the image generated from the first time point of the FID was poorer than the MP‐RAGE image (0.25 mL versus 0.001 mL), the non‐water‐suppressed metabolite‐cycling MRSI and its reconstruction generated a spectroscopic image with structural information similar to that of MPRAGE.

**Figure 3:**
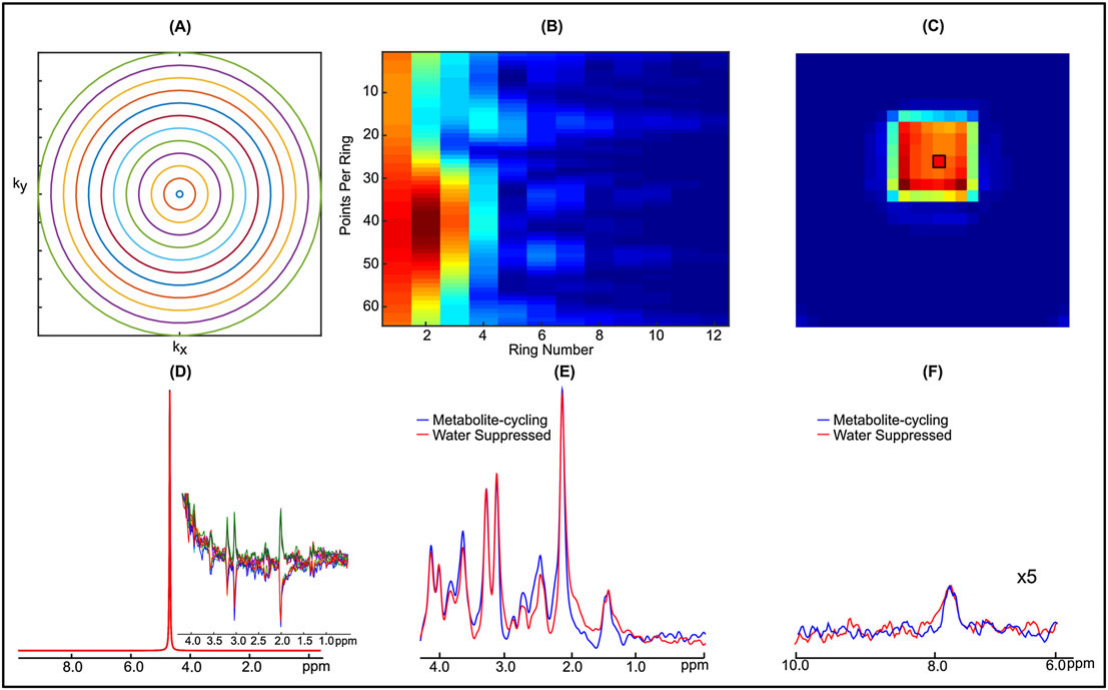
A, Concentric ring *k*‐space trajectory used for the braino phantom. B, *k*‐space data acquired using non‐water suppressed metabolite‐cycling MRSI with following parameters: *N*_ring_ = 12, *N*_p_ring_ = 64, FOV = 240 mm × 240 mm, STEAM localization =65 mm × 65 mm × 20 mm, *T*_R_ = 2 s, *T*_E_ = 14 ms, *T*_M_ = 32 ms, ADC bandwidth =80 kHz, *N*_avg_ = 10 and maximum slew rate = 67.8 mT/m/ms. C, Water image with a final grid of 24 × 24 (2*N*_ring_ × 2*N*_ring_) obtained using the first time point of the water FID. D, Spectra of 10 non‐water‐suppressed water peaks from a voxel taken from the STEAM localized region (black box in C). The subfigure illustrates the effect of asymmetric RF pulses on the upfield spectrum. E,F, Representations of upfield (E) and downfield (F) spectra extracted from a 2 mL voxel (black box in C) from the data acquired using non‐water‐suppressed (blue) and water‐suppressed (red) STEAM localization. The residual water peak was filtered with the HLSVD algorithm. Phantom spectra were line broadened (6 Hz) to match line widths encountered *in vivo*

Figure 3 shows results from the braino phantom. Spectra from a 6 × 6 grid localized by non‐water‐suppressed metabolite‐cycling MRSI (Figure 3C) were acquired using the concentric *k*‐space trajectory (Figure 3A,B). Water spectra of 36 localized voxels of odd and even scans for non‐water‐suppressed metabolite‐cycling MRSI are shown in Figure 3D. The correction factor, *k*, was typically between 1.0007 and 1.0029 for phantom experiments, resulting in a negligible SNR loss.^14^ The upfield spectra edited by the asymmetric RF pulses are highlighted as a subfigure in Figure 3D. An overlaid comparison of representative upfield and downfield spectra acquired by non‐water‐suppressed and water‐suppressed methods is illustrated in Figure 3E,F, respectively. Qualitatively, the spectra match up well. Table 1 gives a quantitative comparison of the two methods listing SNR, estimated LCModel metabolite concentrations and Bland‐Altman reproducibility coefficient. Quantitative analysis of both methods using the ratio of tNAA, Cho, *myo*‐Ins and Lac with respect to Cr resulted in values similar to the specification of the braino phantom.^44^ A strong positive correlation (*r* ^2^ > 0.95, *p* < 0.01) between concentrations obtained from non‐water‐suppressed and water‐suppressed MRSI was found (36 voxels and five metabolites) (Supporting Figure S2). The mean difference in the estimated concentration across metabolites had a small positive bias (mean + SD, 0.035 ± 0.88). The confidence intervals in the limits of agreement defined as the mean concentration/reproducibility coefficient for tNAA (13%), *myo*‐Ins (19%) and Cho (15%) were less than those of Glu (24%) and Lac (32%).

**Table 1:** Bland–Altman statistics for phantom measurements. Mean metabolite concentration ratios with respect to Cr and Bland–Altman reproducibility coefficient of non‐water‐suppressed and water‐suppressed acquisition schemes (mmol) measured using the Bland–Altman method. SNR, the maximum peak‐height divided by the root mean square of residual noise, was calculated using LCModel

	Mean Concentration, Reproducibility Coefficient (mmol)	Phantom Concentration (mmol)
tNAA	12.19, 1.60	12.50
Glu	12.20, 2.90	12.50
tCho	3.08, 0.46	3.00
myo‐iIns	6.88, 1.30	7.50
Lac	3.938, 1.21	5.00
	Non‐Water	Water
	Suppressed	Suppressed
SNR	28.05 ± 3.46	29.33 ± 3.26

**Figure 4:**
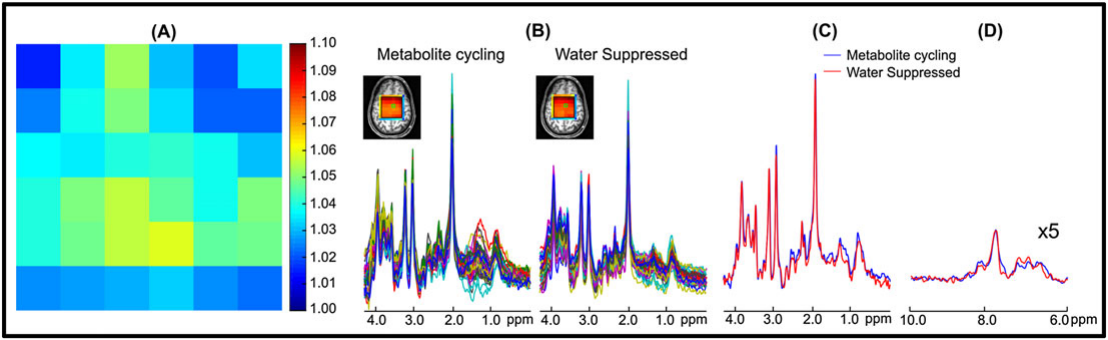
A, Spatial distribution of the correction factor, *k*, in the region of STEAM localization. B, Spectra from localized 6 × 6 voxels acquired using non‐water‐suppressed metabolite‐cycling and water‐suppressed methods without any apodization. Insets show water images overlaid on an anatomical image obtained using the first time point of the water FID. C,D, Representative upfield (C) and downfield (D) spectra are shown extracted from a 2 mL voxel (green box in B). The residual water peak was filtered with the HLSVD algorithm

Figure 4 illustrates *in vivo* MRSI results from a volunteer. The correction factor, *k*, was typically between 1.015 and 1.058 in the localized voxels for *in vivo* measurement, resulting in a negligible SNR loss of less than 0.05% (Figure 4A).^14^ As shown in Figure 4B,C, reasonable agreement was found between the spectra from non‐water‐suppressed and water‐suppressed MRSI spectra in the range from 1.8 to 4.2 ppm in 36 localized voxels (as indicated at the top left of Figure 4B). An overlaid comparison of representative downfield and upfield spectra acquired by non‐water‐suppressed and water‐suppressed methods is illustrated in Figure 4C,D.

**Figure 5:**
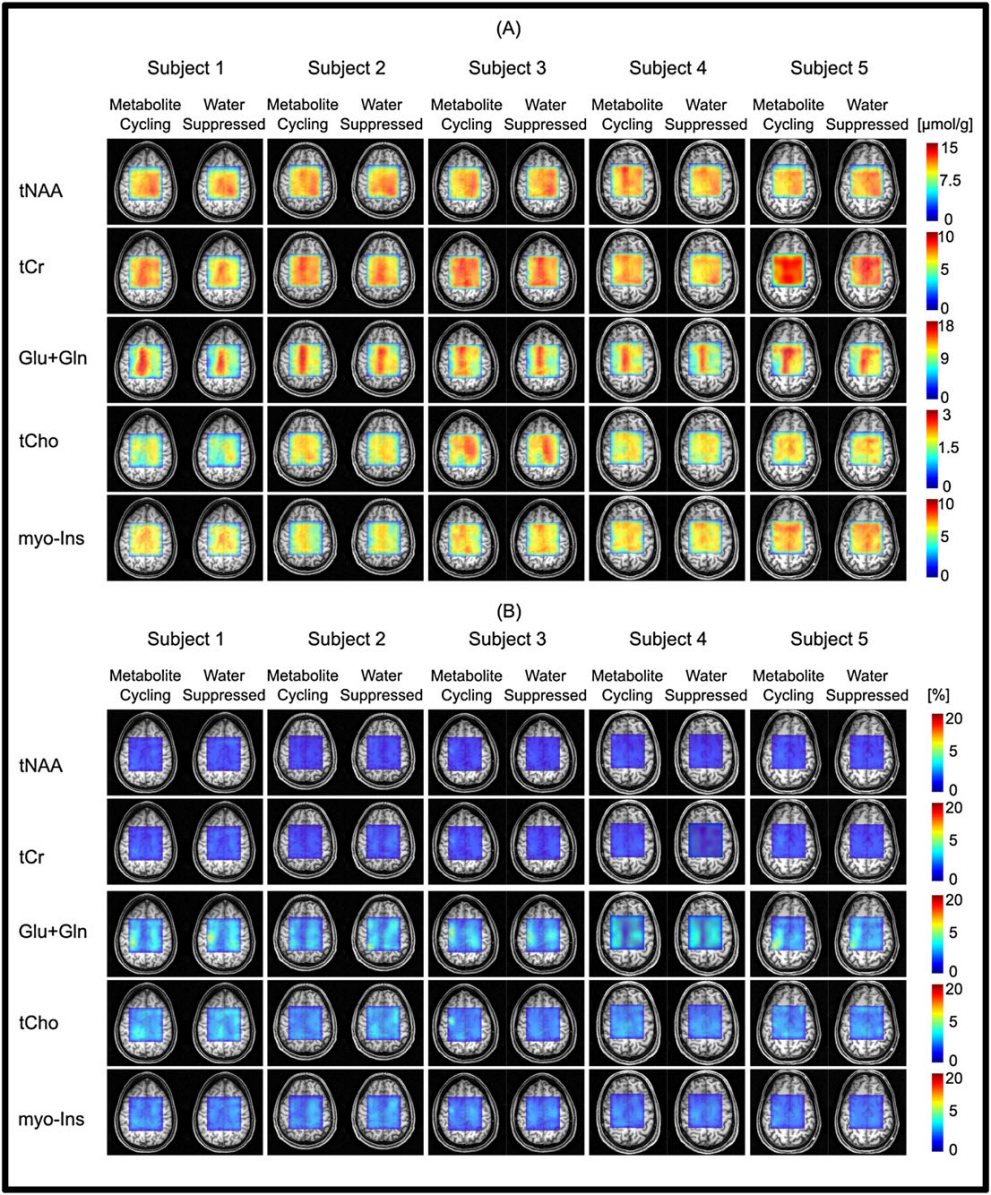
Metabolite and CRLB distribution maps obtained from all subjects. Absolute metabolite concentration (A) and CRLB (B) maps of tNAA, tCr, Glu + Gln, tCho and *myo*‐Ins overlaid on an anatomical image

Figure 5A shows the results across all five subjects of quantification with LCModel. Due to the high spectral quality provided by both techniques, five important brain metabolites could be mapped for all subjects with CRLBs less than 20%. The goodness of the LCModel fit of these metabolites is further visualized by the CRLB maps (Figure 5B) shown for all subjects. CRLB and metabolite maps are complementary to each other for both methods; metabolites with low concentrations (Figure 5A) showed high CRLBs (Figure 5B).

**Figure 6:**
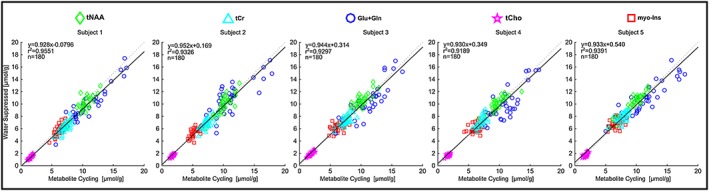
Correlation between concentrations quantified from each localized voxel using non‐water‐suppressed metabolite‐cycling and water‐suppressed MRSI from all subjects. The dashed (grey) and solid (black) lines represent the unity and linear regression lines, respectively

**Figure 7:**
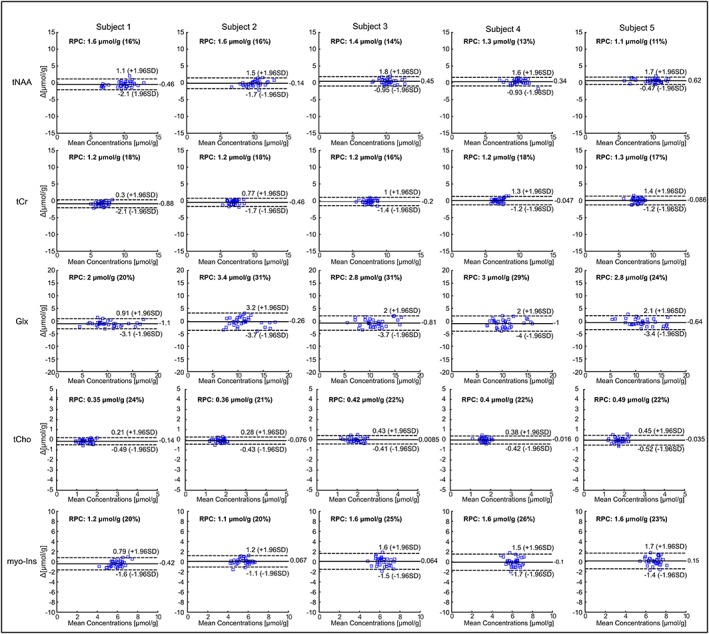
Bland–Altman analysis of *in vivo* measurements. Bland–Altman plots indicate the limits of agreement between metabolite concentrations quantified from non‐water‐suppressed metabolite‐cycling and water‐suppressed MRSI from each subject. The *y*‐axis shows the difference between the two techniques for each localized voxel (water suppressed − non‐water‐suppressed, *Δ*) and the *x*‐axis represents the average of these measures ((water suppressed + non‐water‐suppressed)/2, mean). The dotted lines represent ±1.96 SD with the limits of agreement. The solid line represents the mean bias

The degree to which the two techniques were in agreement was determined by linear regression analysis. A strong positive correlation (*r* ^2^ > 0.91, *p* < 0.01) between concentrations obtained from non‐water‐suppressed and water‐suppressed MRSI was found for five brain metabolites for all subjects (36 voxels and five metabolites) (Figure 6). Although the slope of the regression line was very close to unity (mean slope across all subjects =0.93 ± 0.01), the metabolite concentration estimated using the non‐water‐suppressed metabolite‐cycling MRSI method resulted in slightly higher concentrations. To characterize this bias in more detail, the agreement between the non‐water‐suppressed and water‐suppressed methods was analysed using a Bland‐Altman analysis to plot the difference between the two measurements versus their mean (Figure 7). The mean difference of the tNAA across all subjects had a small positive bias (mean ± SD, 0.16 ± 0.44) whereas the mean difference of tCr, Glu + Gln, tCho and *myo*‐Ins had negative biases (−0.33 ± 0.34, −0.76 ± 0.33, −0.06 ± 0.05 and −0.05 ± 0.22, respectively). The confidence intervals for the limits of agreement (Table 2).are narrower for tNAA (14 ± 2.1% of mean) and tCr (17.8 ± 0.4% of mean), and slightly wider for Glu + Gln (25 ± 5.5% of mean), tCho (22.2 ± 1.1% of mean) and *myo*‐Ins (22.8 ± 2.7% of mean). SNR and linewidths were no different (*p* > 0.05) between the two methods.

**Table 2:** Bland–Altman statistics for *in vivo* measurements. Mean absolute metabolite concentration and Bland–Altman reproducibility coefficient of non‐water‐suppressed and water‐suppressed acquisition techniques (μmol/g) measured using the Bland–Altman method. LW and SNR were calculated using LCModel analysis

Mean Concentration, Reproducibility Coefficient (μmol/g)										
	Subject 1		Subject 2		Subject 3		Subject 4		Subject 5	
tNAA	9.80, 1.60		10.00, 1.60		10.00, 1.40		9.90, 1.30		9.90, 1.10	
tCr	6.70, 1.20		7.00, 1.20		7.50, 1.20		7.00, 1.20		7.80, 1.30	
Glu + Gln	10.00, 2.00		11.00, 3.40		11.00, 2.80		10.00, 3.00		11.00, 2.80	
tCho	1.50, 0.35		1.70, 036		1.90, 0.42		1.70, 0.40		1.80, 0.49	
myo‐Ins	6.00, 1.20		5.50, 1.10		6.30, 1.60		6.20, 1.60		6.90, 1.60	
	Non‐Water	Water	Non‐Water	Water	Non‐Water	Water	Non‐Water	Water	Non‐Water	Water
	Suppressed	Suppressed	Suppressed	Suppressed	Suppressed	Suppressed	Suppressed	Suppressed	Suppressed	Suppressed
Linewidth (Hz)	5.86 ± 0.90	5.87 ± 0.75	6.07 ± 1.06	5.99 ± 0.96	5.87 ± 0.86	5.91 ± −0.74	6.20 ± 0.83	5.80 ± 0.79	5.96 ± 0.98	5.80 ± 0.93
SNR	24.63 ± 3.42	23.50 ± 3.32	27.55 ± 3.16	21.94 ± 3.40	26.55 ± 4.33	29.97 ± −4.33	27.11 ± 3.83	29.88 ± 4.00	24.61 ± 4.33	26.50 ± 5.31

**Figure 8:**
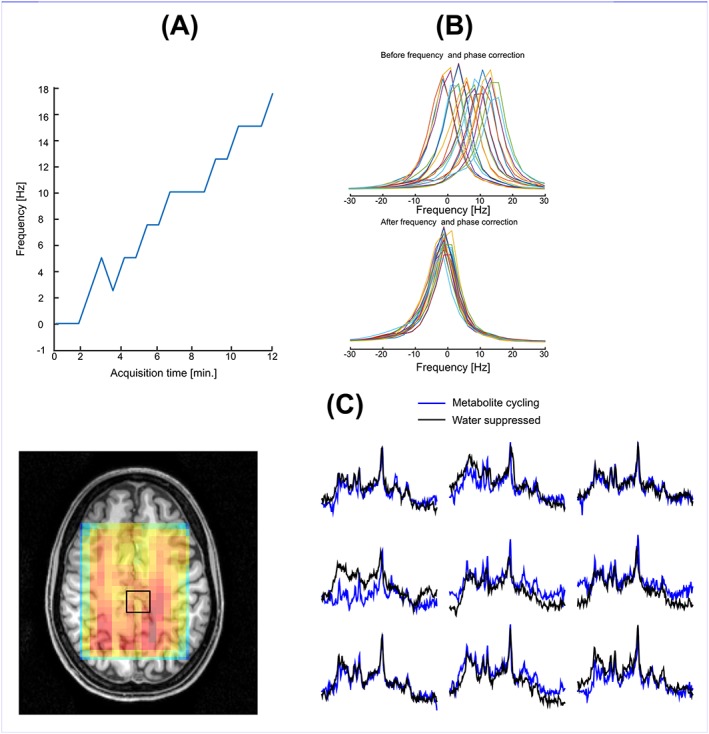
A, Water resonance frequency versus acquisition time. In order to simulate frequency changes due to patient motion and long‐term magnetic field drift, during *in vivo* MRSI measurements transmit and receive frequency offsets were varied from 0 Hz to 18 Hz with a 2 Hz increment for every other MRS image. B, Spectra of 20 non‐water‐suppressed water peaks of one volunteer without (top) and with (bottom) frequency alignment and phase correction. C, Spectra from high resolution (5 mm × 5 mm × 20 mm) using non‐water‐suppressed metabolite‐cycling and water‐suppressed methods. The inset shows the water image overlaid on an anatomical image

Due to the high SNR water peak in the high‐resolution MRSI voxel (~0.5 mL), non‐water‐suppressed metabolite‐cycling MRSI successfully detected the frequency changes induced by the transmit and receive frequency offsets (Figure 8A), whereas water‐suppressed MRSI could not detect these due to poor SNR. Thus, non‐water‐suppressed metabolite‐cycling MRSI enables voxel‐wise single‐scan frequency, phase and eddy current correction of metabolite spectra before averaging (Figure 8B), which resulted in visually discernible improvement in spectral quality as compared with water‐suppressed spectra (Figure 8C).

## 4 DISCUSSION

This study demonstrates that short‐*T*_E_ 2D MRSI data can be obtained using both non‐water‐suppressed metabolite‐cycling and water‐suppressed techniques in the same measurement time as SV‐MRS. We have shown that the non‐water‐suppressed metabolite‐cycling method can produce high‐quality spectra similar to those produced by the water‐suppressed technique. The metabolite concentration values measured using the short‐*T*_E_ MRSI for different tissues were consistent with previous literature values (see later). Finally, high‐resolution non‐water‐suppressed metabolite‐cycling MRSI (~0.5 mL) resulted in a significant improvement in the spectral quality compared with water‐suppressed spectra by using the water peak for voxel‐wise single‐scan frequency, phase and eddy current correction. To our knowledge, this is the first study to have validated the use of non‐water‐suppressed metabolite‐cycling techniques for short‐*T*_E_ MRSI.

In addition to the reduction of sideband artefacts and eddy currents, it has been shown previously that metabolite‐cycling offers improved frequency alignment and phase correction for SV‐MRS in voxels where insufficient SNR does not permit correction of single‐scan acquisitions.^14^, ^15^ In this study, we utilized metabolite‐cycling for the MRSI acquisition within a clinically feasible acquisition time using a concentric ring *k*‐space trajectory. This acquisition scheme allowed us to obtain water and metabolite resonances simultaneously without requiring any additional water‐suppressed measurements. Similarly to the earlier SV metabolite‐cycling studies,^14^, ^15^ the non‐water‐suppressed spectra were used as an internal reference for quantification of the metabolite signals and as a reference to correct residual eddy currents, coil combination and frequency drifts in the *B*_0_ field due to subject motion and/or hardware instability. The metabolite spectra (subtracted spectra) and estimated concentrations were in agreement with the water‐suppressed MRSI acquisitions (Figure 4). Since both non‐water‐suppressed and water‐suppressed MRSI resulted in high spectral quality over the STEAM localization, the single‐scan frequency and phase correction were applied for each voxel in the STEAM localization. However, the high‐resolution MRSI (~0.5 mL), where intrinsic SNR is low, benefited more substantially from the metabolite‐cycling technique due to the high SNR of the water signal used for pre‐processing steps (Figure 8).

The achieved spectral quality allowed reliable quantification of major brain metabolites with a CRLB of less than 20% using LCModel analysis. Concentration distributions of metabolites quantified in this study were in good agreement with previously reported values acquired from the same brain locations^45^ and revealed significant variations between different brain tissues (Figure 5). The findings are in agreement with previous MRS studies of the anatomical locations most similar to the regions presented here. For example, Glu + Gln, tCr and *myo*‐Ins showed higher concentrations in the region of grey matter (GM) compared with white matter (WM).^45^, ^46^, ^47^ The concentration of tNAA had a fairly homogeneous distribution, consistent with previous MRS studies.^45^, ^46^, ^47^ In addition, we found elevated tCho, specifically tCho/tCr, in WM in comparison with GM.^48^

There remain several limitations of the implemented methods. First, although we used a short gap (9.6 ms) between the STEAM excitation and asymmetric adiabatic RF inversion pulse, the long *T*_M_ (32 ms) period of non‐water‐suppressed metabolite‐cycling STEAM MRSI might lead to a magnetization transfer effect on several metabolites.^49^ This can be minimized by incorporating the asymmetric adiabatic RF inversion pulse into the TM period.^16^ Second, metabolite signals closest to the water peak, such as *myo*‐Ins, tCho, Lac and tCr, might be reduced due to imperfections in the chemical‐shift‐selective RF pulses caused by local magnetic field variability. The asymmetric adiabatic RF inversion pulses used in this study were designed to minimize the effect of local magnetic field variability by placing the centre of the transition band (*M*_*z*_ = 0) at the carrier frequency offset by ±60 Hz. This is further supported by the negative concentration biases of tCr, tCho and *myo*‐Ins (Figure 7). indicating that local magnetic field variability induced more signal loss for the water‐suppressed MRSI technique. The signal intensities of these metabolites might be reduced by the water suppression due to magnetization exchange with water.^18^ In addition, these negative concentration biases could also be explained by the shortening of *T*_1_ relaxation times in the 1–4 ppm region when non‐water‐suppressed metabolite‐cycling STEAM MRSI is used.^50^ Furthermore, when larger VOIs are studied, for which the *B*_1_ and *B*_0_ fields will be moderately inhomogeneous at 3 T, resulting in imperfect inversion, utilization of *B*_1_ and higher‐order *B*_0_ shimming might help achieve homogeneous *B*_1_ and *B*_0_ over the entire VOI. Another potential limitation of the implemented metabolite‐cycling technique was higher lipid contamination due to the omission of the OVS pulses, which affects macromolecule resonances in the 0.5 to 2.0 ppm region (Figure 4). Although the metabolite‐cycling technique is a spectral subtraction‐based technique and is vulnerable to subject motion or hardware instability leading to subtraction errors, non‐water‐suppressed MRS images are expected to be used for real‐time motion and scanner drift artefact corrections to minimize these errors. Finally, in this study, we chose to utilize STEAM localization with a penalty of twofold signal loss compared with PRESS or semi‐LASER since short T_E_ values can easily be achieved with lower peak RF pulse powers, yielding a lower specific absorption rate (SAR). This is especially important for potential short‐*T*_R_ high‐resolution MRSI studies in humans, where SAR might be a problem.

In conclusion, we have developed and demonstrated a non‐water‐suppressed metabolite‐cycling MRSI technique that performs robustly on clinical MRI scanners. The proposed novel MRSI technique will be beneficial not only for acquiring simultaneous metabolite and internal reference data, but also for providing calibration information for combining phased‐array coil and data for correcting residual eddy currents, as well as phase and frequency drifts. These advancements can easily be extended to whole brain MRSI via advanced post‐processing and reconstruction techniques.^51^

## Supporting information


**Supporting Figure S1** (a) High resolution T1‐weighted MPRAGE image of the slice studied and (b) the image acquired using non‐water‐suppressed metabolite‐cycling MRSI with parameters N_ring_ = 24, N_p_ring_ = 64, FOV = 200 mm × 200 mm, STEAM localization =115 mm × 115 mm × 10 mm, TR = 1 s, TE = 14 ms, TM = 32 ms, ADC bandwidth =80 kHz, N_avg_ = 2 and maximum gradient slew rate = 168.2 mT/m/ms, mT/m/ms. Water image with a final grid of 48 × 48 (2N_ring_ × 2N_ring_) obtained using the first time point of the water FID.
**Supporting Figure S2** Left: Correlation between concentrations quantified from each localized voxel using non‐water‐suppressed metabolite‐cycling and water‐suppressed MRSI from the braino phantom measurement. Right: Bland–Altman analysis of the braino phantom measurement. Bland–Altman plots indicate the limits of agreement between metabolite concentrations quantified from non‐water‐suppressed metabolite‐cycling and water‐suppressed MRSI from each subject. The y‐axis shows the difference between the two techniques for each localized voxel (water‐suppressed ‐ non‐water‐suppressed, Δ) and the x‐axis represents the average of these measures ((water‐suppressed + non‐water‐suppressed)/2, mean). The dotted lines represent ±1.96 SD with the limits of agreement. The solid line represents the mean bias.
