# Supplementary material for: Non‐water‐suppressed short‐echo‐time magnetic resonance spectroscopic imaging using a concentric ring k‐space trajectory
Source: NMR Biomed. 2017 Mar 8;30(7):e3714. doi: 10.1002/nbm.3714 (PMC5485000; doi:10.1002/nbm.3714)
Supplement: Supplementary file 1 — Supporting Figure S1 (a) High resolution T1‐weighted MPRAGE image of the slice studied and (b) the image acquired using non‐water‐suppressed metabolite‐cycling MRSI with parameters Nring = 24, Np_ring = 64, FOV = 200 mm × 200 mm, STEAM localization =115 mm × 115 mm × 10 mm, TR = 1 s, TE = 14 ms, TM = 32 ms, ADC bandwidth =80 kHz, Navg = 2 and maximum gradient slew rate = 168.2 mT/m/ms, mT/m/ms. Water image with a final grid of 48 × 48 (2Nring × 2Nring) obtained using the first time point of the water FID. Supporting Figure S2 Left: Correlation between concentrations quantified from each localized voxel using non‐water‐suppressed metabolite‐cycling and water‐suppressed MRSI from the braino phantom measurement. Right: Bland–Altman analysis of the braino phantom measurement. Bland–Altman plots indicate the limits of agreement between metabolite concentrations quantified from non‐water‐suppressed metabolite‐cycling and water‐suppressed MRSI from each subject. The y‐axis shows the difference between the two techniques for each localized voxel (water‐suppressed ‐ non‐water‐suppressed, Δ) and the x‐axis represents the average of these measures ((water‐suppressed + non‐water‐suppressed)/2, mean). The dotted lines represent ±1.96 SD with the limits of agreement. The solid line represents the mean bias. [file NBM-30-na-s001.docx]

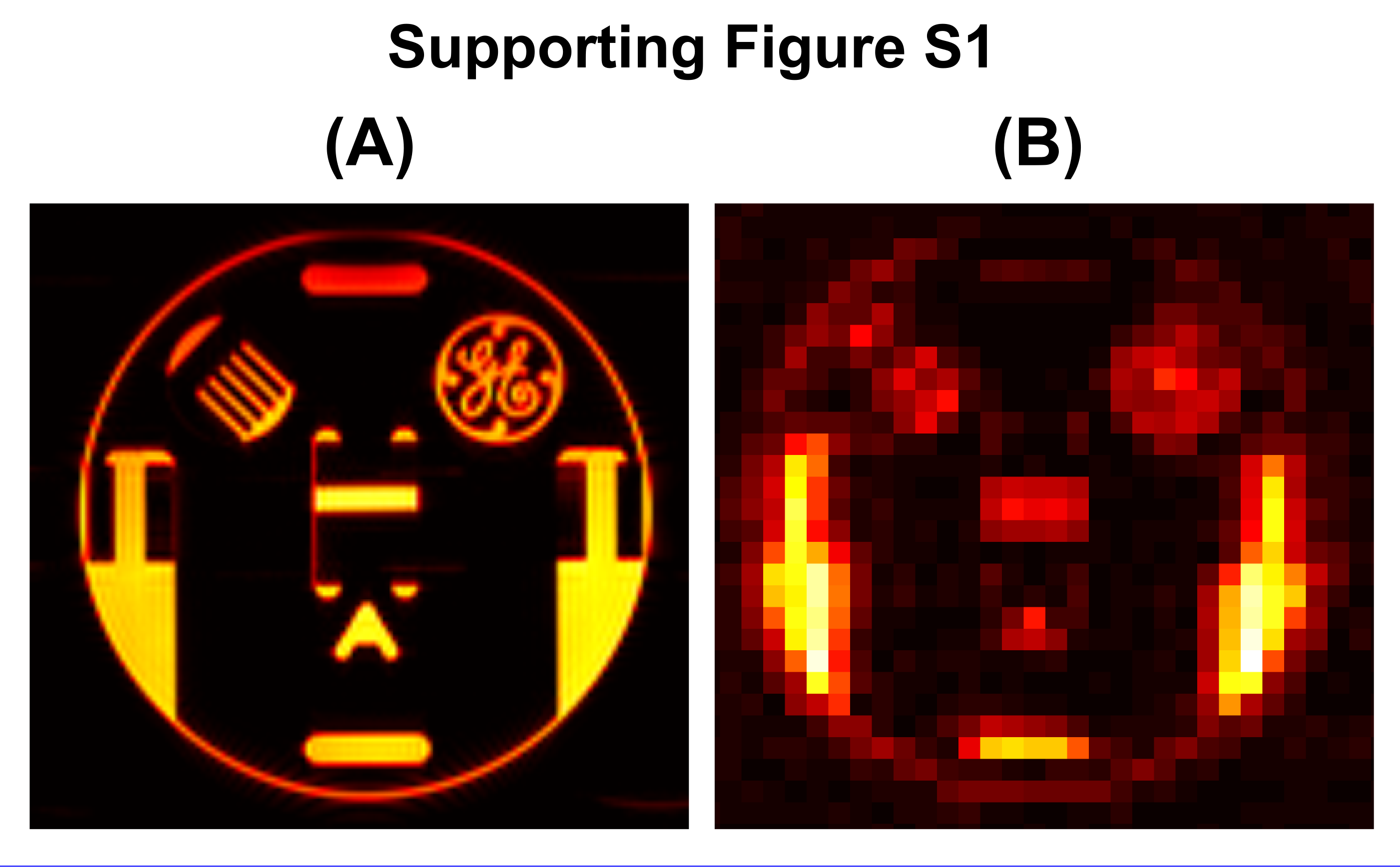


**Supporting Figure S1** (a) High resolution T1-weighted MPRAGE image of the slice studied and (b) the image acquired using non-water-suppressed metabolite-cycling MRSI with parameters N_ring_ = 24, N_p_ring_ = 64, FOV = 200 mm x 200 mm, STEAM Localization = 115 mm x 115 mm x 10 mm, TR = 1 s, TE = 14 ms, TM = 32 ms, ADC bandwidth = 80 kHz, N_avg_ = 2 and maximum gradient slew rate = 168.2 mT/m/ms, mT/m/ms. Water image with a final grid of 48 x 48 (2N_ring_ x 2N_ring_) obtained using the first time point of the water FID.


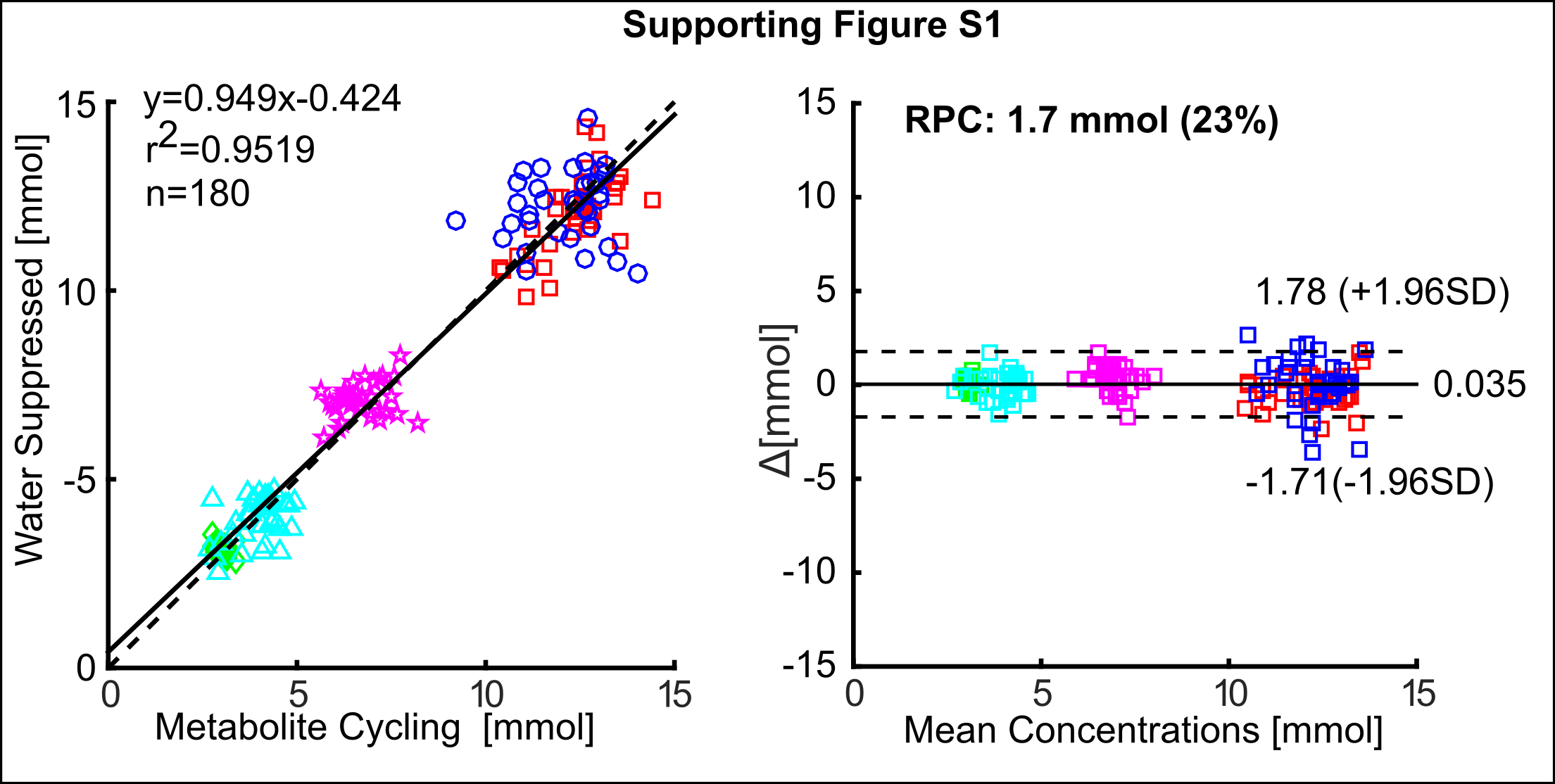


**Supporting Figure S2** Left: Correlation between concentrations quantified from each localized voxel using non-water-suppressed metabolite-cycling and water-suppressed MRSI from the braino phantom measurement. Right: Bland-Altman analysis of the braino phantom measurement. Bland-Altman plots indicate the limits of agreement between metabolite concentrations quantified from non-water-suppressed metabolite-cycling and water-suppressed MRSI from each subject. The y-axis shows the difference between the two techniques for each localized voxel (water-suppressed – non-water-suppressed, Δ) and the x-axis represents the average of these measures ((water-suppressed + non-water-suppressed)/2, mean). The dotted lines represent ±1.96 SD with the limits of agreement. The solid line represents the mean bias.
